# Re-Examining the Dimensionality of a Disability Assessment Tool Using Exploratory Structural Equation Modeling (ESEM): A Preliminary Study in Low Back Pain

**DOI:** 10.3390/jcm14238551

**Published:** 2025-12-02

**Authors:** Serhat Hayme, Derya Gökmen, Şehim Kutlay, Ayşe A. Küçükdeveci

**Affiliations:** 1 Department of Biostatistics and Medical Informatics, Faculty of Medicine, Erzincan Binali Yıldırım University, Erzincan 24100, Turkey; shayme@erzincan.edu.tr; 2 Department of Biostatistics, Faculty of Medicine, Ankara University, Ankara 06290, Turkey; 3 Department of Physical Medicine and Rehabilitation, Faculty of Medicine, Ankara University, Ankara 06100, Turkey; skutlay@medicine.ankara.edu.tr (Ş.K.); akucuk@medicine.ankara.edu.tr (A.A.K.)

**Keywords:** low back pain, disability assessment, ESEM, CFA, multidimensionality, ICF

## Abstract

**Background**: Low back pain (LBP) profoundly impacts daily life, requiring assessment tools that capture its complex effects on the body and mind. This study explores a measurement tool designed to assess LBP disability, testing whether Exploratory Structural Equation Modeling (ESEM) better reveals its multidimensional nature compared to Confirmatory Factor Analysis (CFA). **Methods**: We analyzed data from 266 LBP patients using CFA and ESEM. The tool, developed from the items from existing scales, included 99 questions on body functions, activities, and participation. Using Mplus 8 software, we compared model fit and item connections. **Results**: Two main factors; “Body Functions” and “Activity-Participation” identified by CFA were tested using ESEM. While ESEM had slightly better fit compared to CFA model, many items linked across both factors which shows how pain and emotions overlap with daily activities. These results align with the International Classification of Functioning, Disability and Health (ICF) and reflect LBP’s broad impact. **Conclusions**: ESEM offers a broader understanding of LBP’s multidimensional nature compared with CFA, guiding clinicians to create a holistic management approach that address physical and psychosocial challenges. This preliminary study supports the use of ESEM in disability research, demonstrating its usefulness in identifying the multifaceted nature of LBP, therefore providing a broader perspective for assessment and management.

## 1. Introduction

Low back pain (LBP) is one of the most common musculoskeletal problems globally, necessitating urgent action to address its widespread impact on health systems and economies [1]. It affects a large portion of the population at some point in their lives and can have a significant impact on an individual’s quality of life. The economic burden of LBP is substantial, both at the societal and individual levels, with significant implications for healthcare systems and workforce productivity [2]. The prevalence of LBP is high, with a global age-standardized prevalence rate of 7460 per 100,000 in 2020 [3]. It remains the leading cause of disability worldwide, contributing significantly to disability-adjusted life years (DALYs) and posing a major public health challenge.

The assessment of patients with LBP is of crucial importance for the effective planning and monitoring of therapeutic interventions. A variety of instruments are available for outcome assessments [4] and standardized ‘core sets’ based on the International Classification of Functioning, Disability and Health (ICF) have been introduced with a view to enhancing consistency in evaluation. These core sets facilitate the integration of physical and psychosocial domains in clinical practice [5,6].

The integration of multidimensional approaches that address both physical and psychosocial factors provides a more complete picture of the patient’s condition, ensuring that the treatment plan is not just focused on pain relief but also on improving activities, participation and quality of life. Research has shown that LBP profoundly affects patients’ daily functioning and emotional well-being, necessitating comprehensive assessment tools [7].

There are many assessment tools that have been developed for outcome measurement in LBP [4]. The choice of outcome measures for evaluating LBP is indeed a challenge for clinicians, and this issue is exacerbated by the wide variety of available tools, each with its own set of psychometric properties, terminology, and methodological approaches. Variability in clinical assessments further complicates standardized evaluation, highlighting the need for robust outcome measures [8,9].

Measurement tools are utilized to acquire information about the trait levels of individuals in daily life and scientific research across various disciplines. When they consist of heterogeneous items, it is logical to evaluate the data using multidimensional models [10]. Psychometric multidimensionality refers to the idea that the items may be associated with more than one source of true score variance (more than one dimension). Psychometric multidimensionality has two structural sources: (1) The fallible nature of items as if they were perfect indicators of a single construct. This situation often occurs in measurement tools that evaluate conceptually related and partially overlapping constructs. (2) The hierarchical nature of the assessed constructs. This occurs when all items have a meaningful relationship with the structure they are intended to measure [11].

The presence of the first source of psychometric multidimensionality is assessed through the comparison of confirmatory factor analysis (CFA) and exploratory structural equation modeling (ESEM) solutions. In CFA, there is a constraint that the items should only be related to their target factors and unrelated to non-target factors. This constraint leads to the evaluation of items as perfect indicators of the relevant dimension. As this constraint may not be suitable for measurement tools with multidimensional structures (constructs that can be related to each other), ESEM has been widely used in recent years to overcome these issues [12,13].

While SEM combines CFA and path analysis [14], ESEM integrates Exploratory Factor Analysis (EFA) into the SEM framework [12,13]. It was developed to overcome the rigidity of traditional SEM by allowing cross-loadings (i.e., items can load on multiple factors), but still within a structural equation context that lets to test relationships among latent variables. In ESEM, the advantage of EFA—allowing all items in the measurement tool to be related to all factors—can be combined with the advantages of CFA which evaluates model fit using fit indices, comparing different models, testing structural models using factor scores, measuring invariance. This integration makes ESEM particularly suitable for assessing complex, multidimensional constructs in health research. Additionally, since ESEM permits all items to be related to all factors, the factor correlations obtained from ESEM are lower than those obtained from CFA. This situation implies that the structural relationships between factors can be more accurately determined [12].

Although EFA and CFA are commonly used methods for evaluating the dimensionality of measurement tools in the literature, the assessment of multidimensionality of the scales through ESEM models is a relatively newer approach. Applications of this method on outcome instruments used in the fields of psychology and psychiatry have played a triggering role in promoting the use of this method in the healthcare field. To the best of our knowledge, there is no study in the literature in which assessment tools related to LBP were evaluated with ESEM. The aim of this preliminary study is to examine the dimensionality structure of an assessment tool developed to evaluate the disability of LBP patients with the ESEM model.

## 2. Materials and Methods

### 2.1. Participants

The study utilized data from a previously completed study [5] that was conducted as a PhD thesis [15]. The data was originally collected between 2005 and 2008 and obtained from 266 patients with LBP at the Department of Physical Medicine and Rehabilitation (PMR), Ankara University Medical Faculty. The study was approved by the local research and ethics committee (2008/138-3999). The present study, conducted within the scope of another doctoral thesis completed in 2020–2021 [16], re-analyzed this existing dataset using advanced psychometric models (CFA and ESEM).

### 2.2. Study Design and Materials

The assessment tool used in this study was based on the previous study mentioned above [5,15]. During that study, the items intended to be included in the measurement tool were determined by examining existing scales used in the evaluation of patients with LBP. These scales assess “body functions”, “activities” and “participation” in the context of the ICF framework and the ICF core set for LBP [17,18]. As part of this process, data obtained from the measurement tool created for determining the disability levels of patients with LBP included the responses of the patients to 99 questions (with response categories ranging from 2 to 6). These questions were derived from the items of the World Health Organization Disability Assessment Schedule II (WHODAS-II), Oswestry Disability Index (ODI), Roland Morris Disability Questionnaire (RMDQ), and the Nottingham Health Profile (NHP) scales, with the RMDQ and the ODI commonly used for measuring disability in LBP [15,19,20,21]. As the main aim of the previous study [5,15] was to perform computerized adaptive testing, the instrument used in that study was considered as an item bank with two dimensions: body functions, and activity-participation. While the items in body functions dimension were related to the pain, sleep, cognitive and emotional aspects of health, activity-participation dimension included items related with mobility, self-care activities, domestic and social life.

### 2.3. Data Analysis

In this study, the presence of the fallible nature of the items as perfect indicators of a single construct was assessed using two-factor CFA and ESEM models, leveraging modern factor analytic techniques to capture complex multidimensional structures [22]. The use of two-factor models is based on the previous study [5], which demonstrated that the 99 items have a two-factor structure. A positive factor loading in CFA/ESEM indicates that an item contributes to some degree to the factor and a negative loading indicates that its absence contributes to some degree to the factor. In the two-factor CFA model, each item was allowed to load on its target factor, while its loading on the non-target factor was assumed to be zero. In the two-factor ESEM model, the target and non-target loadings were obtained using target (oblique) rotation, ensuring that the factors were related. This approach provides a balance between exploratory flexibility and confirmatory interpretability, aligning with the theoretical assumption that disability-related dimensions are interrelated.

Within this context, the CFA and ESEM models were compared based on model fit indices, factor inter-correlations, and factor loading patterns. In the examination of model fit, different fit indices were utilized [23]. As each type of fit index provides different information about model fit; therefore, researchers are advised to consider at least one fit index from each category when evaluating the adequacy of their models [24]. In this study, model fit was considered adequate when both selected indices fell within the recommended ranges (Table 1). Also, item loadings (with standard errors), factor inter-correlations (with standard errors) and item R^2^ values were calculated for both CFA and ESEM models. If the solution from the ESEM model demonstrates better model fit and yields lower factor inter-correlations and statistically significant cross-loadings (when considering the positive and larger loadings on the target factors than cross-loadings), it will provide evidence to determine whether the ESEM model is a better fit compared to the CFA model.

In order to examine reliability, composite reliability (CR) and factor score determinacy (FSD) coefficients were calculated. CR coefficients for each factor were derived from the standardized ESEM loadings and residual variances. In accordance with established guidelines, CR values ≥0.70 were considered acceptable and values ≥0.80 were regarded as indicative of good internal consistency. Additionally, FSD coefficients were obtained directly from the Mplus output to assess the accuracy and stability of estimated factor scores. FSD values ≥0.80 indicate acceptable determinacy, whereas values ≥0.90 reflect highly reliable factor score estimation [25].

The data analysis was conducted using Mplus 8 version. In the data analysis, the Weighted Least Squares Mean and Variance Adjusted (WLSMV) estimation method was employed. This method is robust against non-normal distributions in the data and is suitable for datasets with categorical or ordinal variables regardless of the number of categories. The complete Mplus input syntax is provided in Supplementary Materials (File S1) for full reproducibility.

## 3. Results

### 3.1. Sample Characteristics

Out of 266 patients, 43 (16.2%) were male, and 223 (83.8%) were female. The mean (±standard deviation) age of the patients was 52.2 (±12.5) years. The mean duration of symptoms was 8.24 years, ranging from a minimum of 0.08 years to a maximum of 40 years.

### 3.2. The Distribution of Items to Factors

The distribution of items across factors, as analyzed in previously mentioned study (5), resulted in 40 items belonging to the “Body Functions” factor and 59 items belonging to the “Activity-Participation” factor. During the examination of the presence of the fallible nature of items as if they were perfect indicators of a single structure, the CFA and ESEM models were compared based on model fit indices, factor inter-correlations and factor loading patterns.

Although the ESEM model [CFI = 0.904; TLI = 0.900; RMSEA = 0.038 (95% CI: 0.035–0.040)] and the CFA model [CFI = 0.900; TLI = 0.898; RMSEA = 0.038 (95% CI: 0.036–0.040)] did not have a significant difference in terms of fit indices, the ESEM model demonstrated slightly better fit to the data. The final decision was given after evaluating factor inter-correlations and factor loadings. During this stage, when examining the factor inter-correlations, the correlation value obtained from the ESEM model (r = 0.547, SE =0.040, *p* < 0.001) was found to be lower than the value obtained from the CFA model (r = 0.643, SE = 0.040, *p* < 0.001). The reason for this difference is that in the CFA model, due to the constraint of cross-loadings being set to zero, the factor correlations were obtained higher than they actually were. In the final stage, the CFA and ESEM models were evaluated in terms of factor loading patterns using standardized factor loadings (Table 2).

The ESEM model clearly revealed that items were associated not only with their target factors but also with non-target factors, showcasing the multidimensional nature of the assessment tool. For instance, the wd1.2 item (remembering to do important things) from the WHODAS-II and o7 item (the duration of sleep disturbed by back pain) from the ODI, which primarily loaded on the “Body Functions” factor (λ = 0.360, *p* < 0.001; λ = 0.459, *p* < 0.001), also showed a statistically significant cross-loading on the “Activity-Participation” factor (λ = 0.265, *p* < 0.001; λ = 0.225, *p* < 0.01). Conversely, the rm21 item (I avoid heavy jobs around the house because of my back) from the RMDQ and n26 item (I soon run out of energy) from the NHP with a target loading on the “Activity-Participation” factor (λ = 0.457, *p* < 0.001; λ = 0.454, *p* < 0.001), exhibited a notable non-target loading on the “Body Functions” factor (λ = 0.229, *p* < 0.05; λ = 0.316, *p* < 0.01). In total, 22 items from the “Body Functions” factor (e.g., wd1.2, o7) and 19 items from the “Activity-Participation” factor (e.g., rm21, n26) demonstrated statistically significant cross-loadings on their respective non-target factors. These cross-loadings align with the ICF framework, reflecting how items related to domains such as communication, social activities, or energy levels span both Body Functions and Activity-Participation constructs. The presence of these prominent and statistically significant non-target loadings underscores that items are not perfect indicators of a single construct, highlighting the strength of ESEM in capturing the complex interplay of physical and psychosocial factors in LBP compared to the more restrictive CFA approach. Item-level R^2^ values ranged from 0.19 to 0.81 for “Body Functions” factor and from 0.04 to 0.88 for “Activity–Participation” factor, indicating varying levels of explained variance across items.

In the CFA model, the factor loadings for the “Body Functions” factor’s 40 items and the “Activity-Participation” factor’s 59 items were all positive and statistically significant (>0.30) on their respective target factors. The descriptive statistics for the factor loadings in the CFA model were obtained as |Λ| = 0.391–0.870 with a mean of 0.684 for the “Body Functions” factor, and |Λ| = 0.210–0.900 with a mean of 0.665 for the “Activity-Participation” factor.

In the ESEM model, all 40 items for the “Body Functions” factor had positive and statistically significant factor loadings. Among these items, two had medium-sized loadings (0.2 < λ < 0.3), and 38 items had high (>0.30) loadings. Similarly, for the “Activity-Participation” factor, 58 out of the 59 items had positive and statistically significant factor loadings. Among these items, one had a small loading (0.1 < λ < 0.2), and 57 items had high (>0.30) loadings. The descriptive statistics for the factor loadings in the ESEM model were obtained as |Λ| = 0.254–0.962 with a mean of 0.626 for the “Body Functions” factor, and |Λ| = 0.149–1.000 with a mean of 0.624 for the “Activity-Participation” factor.

Based on the factor-specific target loadings, it was observed that the factor loadings obtained from the ESEM model were generally lower than those obtained from the CFA model. This discrepancy is due to the ESEM model’s allowance for cross-loadings, which allows the items to be explained by both their target and non-target factors, leading to lower factor loadings overall.

In the context of the ESEM model, items that are loaded on both factors by statistical significance are also evaluated in terms of clinical relevance by the two authors who are experts on disability evaluation (A.A.K and Ş.K). When examining the cross-loadings of the items onto non-target factors, it was found that out of the 40 items belonging to the “Body functions” factor, 22 items had statistically significant positive/negative cross-loadings on the “Activity-Participation” factor. Only 11 of 22 items were found to be clinically relevant. For the 59 items belonging to the “Activity-Participation” factor, 19 items had statistically significant positive/negative cross-loadings on the “Body Functions” factor. However, 17 of 19 items were clinically relevant. Items loaded on both factors statistically and clinically are written in bold in Table 2. Although 22 items of the “Body Functions” factor and 19 items of the “Activity-Participation” factor had statistically significant cross-loadings, the factor loadings of all the items on their target factors were higher than their loadings on non-target factors. Descriptive statistics for the cross-loading values of the items for the “Body Functions” and “Activity-Participation” factors were obtained as |Λ| = 0.004–0.378 with a mean of 0.136, and |Λ| = 0.002–0.393 with a mean of 0.166, respectively.

In the ESEM model, all items belonging to the “Body Functions” factor, except one item from the “Activity-Participation” factor, were found to be statistically significant. Additionally, a considerable number of items in this model showed statistically significant relationships with non-target factors, indicating the presence of the item’s fallible nature. Overall, the positive factor loadings on the target factors, the presence of a limited number of high cross-loadings, and the high selectivity of both factors indicate that the items have a good fit to their respective target factors.

When the reliability and precision indices were calculated from the two-factor ESEM model, composite reliability (CR) was 0.96 for the “Body Functions” factor and 0.95 for the “Activity–Participation” factor. Factor score determinacy (FSD) coefficients computed in Mplus were 0.93 for the “Body Functions” factor and 0.86 for the “Activity–Participation” factor. Although the determinacy of the Activity–Participation factor was slightly below the recommended threshold of 0.90, both values indicate adequate factor score stability, supporting the interpretability and applicability of the ESEM-derived factor scores in clinical and research settings.

## 4. Discussion

In this study, the dimensionality of the assessment tool, which has a two-dimensional structure as “Body Functions” and “Activity-Participation” developed to determine the disability levels of patients with LBP, was evaluated with the ESEM method. The psychometric properties of this instrument were previously demonstrated using CFA (allowing each item to load on only one factor; setting cross-loadings to zero) and it was found to have a two-dimensional structure as “Body Functions” and “Activity-Participation” [5,15]. However, with the introduction of ESEM and its capacity to model cross-loadings in the context of correlated dimensions, a re-evaluation of the instrument’s dimensional structure has become warranted. Clarifying this structure is essential for clinicians to accurately interpret the distinct domains of disability assessed by the scale. Accordingly, the results of CFA and ESEM were evaluated to assess the fallible nature of the items for this instrument.

When the two-factor structure identified in the previous study [5,15] through a CFA model was re-examined using the ESEM model, it was observed that items’ target loadings on the “Body Functions” factor also exhibited cross-loadings on the “Activity-Participation” dimension. Analyzed in terms of their alignment with the ICF framework, these items included domains such as understanding & communicating, getting along with others, sleep, activity-related pain and emotional reactions & social isolation. On the other hand, when examining items with target factor loadings on “Activity-Participation” factor that also showed cross-loadings on the “Body Functions” factor, it was found that these included domains such as household activities, pain severity/intensity, daily activities and energy level. Notably, within the ICF classification, some of these items correspond to both Body Functions and Activity & Participation domains. However, if we consider the CFA model, those items would only correspond to one domain, either Body functions or Activity & Participation. Comparative studies of LBP scales, such as the ODI and RMDQ, further support the need for flexible psychometric models to capture such multidimensionality [26].

The presence of the “fallible nature of items as perfect indicators of a single structure” is assessed by comparing CFA and ESEM solutions. In CFA, there is a constraint that the items are only related to the target factors and unrelated to non-target factors. However, this constraint becomes a significant limitation, especially for assessment tools with multidimensional structures and potentially interrelated constructs. This limitation can lead to the evaluation of items as perfect indicators of the intended factor, even though they may be related to multiple factors, resulting in higher estimated correlations between factors and compromising the discriminant validity of the measurement tool. Additionally, studies have shown that when testing the dimensional structure of multidimensional measurement instruments with CFA, the model fit is rarely within acceptable limits. In CFA, the assumption that each item is only related to the intended structure becomes a challenging constraint, especially in social sciences, health sciences, and education where interrelated constructs are commonly encountered. To address these limitations, ESEM, which combines the advantages of EFA and CFA methods, has become widely used in the literature for assessing psychometric multidimensionality in recent years [13,22].

The comparison between CFA and ESEM results shows that when the fit indices and parameter estimates of the CFA are not significantly different from those of the corresponding ESEM model, researchers may prefer the CFA model as it is a more parsimonious model. However, systematic reviews of LBP outcome measures advocate for models such as ESEM to better account for interrelated constructs [27]. Therefore, when the fit of the ESEM model is better, the estimated factor correlations are lower and small/easily interpretable cross-loadings are present, the ESEM model should be preferred. In this study, when comparing CFA and ESEM results, it was shown that the cross-loadings forced to be zero in CFA were found to be significant in the ESEM context, both in terms of their magnitude and statistical significance. As a supporting finding, the presence of cross-loadings led to lower factor correlations. Similar results were found in chronic pain assessment, where ESEM’s ability to capture cross-loadings between pain intensity, emotional status, and functional limitations enabled clinicians to develop more holistic treatment plans that address both physical and psychosocial factors [27].

In a study conducted by Fresno et al., it was reported that there were numerous studies and different results regarding the factor structure of the “Post-Traumatic Stress Disorder” scale via CFA in the literature. They observed that this was mainly due to the presence of cross-loadings, overlapping of different factors (high correlation values), and even the presence of a global factor. Thus, they aimed to find new evidence regarding the factor structure of the scale using CFA and ESEM. All six CFA models tested showed good fit, and high factor correlations were obtained in all of these models. The ESEM model demonstrated better fit compared to these six models [28].

In cases where there is no hierarchical structure expectation in the evaluation of the factor structure of the measurement instruments, the most commonly used methods are EFA and CFA. However, it is recommended to use the ESEM approach, which integrates the advantages of these two methods, for determining the factor structure.

In the assessment of psychometric multidimensionality of the measurement instruments, the alternative models tested should be evaluated in terms of revealing multidimensionality sources and appropriateness, while taking into account the clinical approach in addition to statistical model fit and parameter estimates. The results obtained from this study show that the clinician’s a priori knowledge and expectations regarding the dimensionality structure of the items in the assessment tool have an important role in the selection of the psychometric model to be used. Understanding minimal clinically important differences in LBP assessments further informs the selection of appropriate psychometric models [29].

The ESEM model is considered to be especially useful in determining the potential bias in evaluating items as perfect indicators of a single structure, particularly when dealing with complex multidimensional instruments. Standardized methodologies, such as COSMIN, further validate the use of advanced psychometric models in health research [30].

As these integrated models have been recently introduced and used, it is important to systematically apply them in the examination of the multidimensionality structure of many complex measurement instruments. As the 22 items of the “Body Functions” factor and 19 items of the “Activity-Participation” factor had statistically significant cross-loadings in ESEM, the “Body Functions” factor is now represented with 59 items instead of 40 items found in CFA in the previous study. Similarly, “Activity-Participation” factor consists of 81 items instead of 59 items found in CFA. In this context, it is important not to narrow down the scope of the measurement instrument in order to ensure unidimensionality. The clinician’s perspective may be subjective, but using ESEM allows for more objective and comprehensive evaluation for the clinician by permitting the cross-loadings.

The current study did not examine measurement invariance or differential item functioning (DIF) across sex or age groups due to the sample composition and exploratory purpose of the analysis. Future studies using larger and more balanced samples are needed to test the configural, metric, and scalar invariance of the proposed factor structure to confirm its generalizability across demographic subgroups.

There are three limitations of this study. Firstly, due to the mean age and gender distribution of this sample, the results cannot be generalized to the LBP population. Secondly, the hierarchical nature of disability was not assessed via CFA or ESEM. In the literature, there is a widespread occurrence of studies demonstrating the preference for using ESEM models in hierarchical models to integrate different solution approaches when both sources of multidimensionality are present. Integrated assessment approaches for LBP further highlight the utility of such models in clinical practice [31,32]. In this context, it is necessary to test whether the two-dimensional structure obtained in future studies falls under a hierarchical dimension. Thirdly, the relatively small sample size (266) may have limited the stability of the polychoric correlation estimates and the generalizability of the results. Future studies with larger and more diverse samples are needed to validate the factor structure more robustly.

## 5. Conclusions

In conclusion, slightly better model fit, lower correlation values, lower factor loadings on the target factors, presence and significance of cross-loadings indicate that the ESEM model provides a better solution than the CFA model in the evaluation of the assessment tool developed for LBP. ESEM models should also be taken into consideration when assessing the dimensionality structure of the assessment scales. Therefore, this study will make a significant contribution to the use of ESEM in health research, as it provides a more flexible way to assess the dimensionality of measurement instruments. To the best of our knowledge, in the current literature, no article assessing any measurement tool for evaluating the disability of LBP patients within ESEM perspective has been found. Thus, this preliminary study can be thought as a first article for ESEM application in LBP. Given the widespread and complex nature of LBP, addressing this prevalent health condition in the context of evaluation requires a multidimensional approach.

## Figures and Tables

**Table 1:** Fit indices and acceptable thresholds.

Fit indices Category	Value	Evaluation
Parsimony-Adjusted Fit	Root Mean Square Error of Approximation (RMSEA) with its 95% confidence interval (CI)	≤0.06—Good fit 0.06–0.08—Acceptable fit 0.08–0.1—Mediocre fit >0.1 Poor fit
Comparative/Incremental Fit	Comparative Fit Index (CFI) Tucker–Lewis Index (TLI)	≥0.95—Good fit 0.90–0.95—Acceptable fit <0.90—Poor fit

**Table 2:** Standardized loadings with (SE)’s and Item R^2^ for CFA and ESEM Solutions.

Items	Two-Factor CFA			Two-Factor ESEM		
	Item Loadings (SE) for Body Functions	Item Loadings (SE) for Activity-Participation	Item R^2^	Item Loadings (SE) for Body Functions	Item Loadings (SE) for Activity-Participation	Item R^2^
**wd1.1—In the last 30 days, how much difficulty did you have in concentrating on doing something for ten minutes?**	0.615 *** (0.045)		0.378	0.403 *** **(0.071)**	0.245 ** (0.074)	0.331
**wd1.2—remembering to do important things?**	0.603 *** (0.045)		0.363	0.360 *** **(0.063)**	0.265 *** (0.060)	0.304
**wd1.3—analyzing and finding solutions to problems in day to** **day life?**	0.714 *** (0.039)		0.510	0.460 *** **(0.063)**	0.288 *** (0.060)	0.440
**wd1.4—learning a new task, for example, learning how to get to a new place?**	0.509 *** (0.058)		0.259	0.299 *** **(0.066)**	0.224 *** (0.064)	0.213
wd1.5—generally understanding what people say?	0.672 *** (0.043)		0.452	0.505 *** **(0.064)**	0.198 ** (0.065)	0.403
wd1.6—starting and maintaining a conversation?	0.670 *** (0.047)		0.449	0.519 *** **(0.068)**	0.184 * (0.075)	0.408
wd4.1—dealing with people you do not know?	0.626 *** (0.064)		0.392	0.716 *** **(0.064)**	−0.073 (0.077)	0.461
wd4.2—maintaining a friendship?	0.391 *** (0.075)		0.153	0.679 *** **(0.075)**	−0.289 ** (0.099)	0.329
wd4.3—getting along with people who are close to you?	0.591 *** (0.06)		0.349	0.652 *** **(0.067)**	−0.044 (0.077)	0.396
wd4.4—making new friends?	0.610 *** (0.062)		0.372	0.857 *** **(0.058)**	−0.249 ** (0.078)	0.563
wd6.3—In the last 30 days, how much of a problem did you have living with dignity because of the attitudes and actions of others?	0.565 *** (0.058)		0.319	0.581 *** **(0.066)**	0.011 (0.07)	0.344
**o7-Sleeping**	0.646 *** (0.039)		0.418	0.459 *** **(0.062)**	0.225 ** (0.067)	0.374
**rm13—My back is painful almost all of the time.**	0.764 *** (0.057)		0.583	0.460 *** **(0.079)**	0.341 *** (0.081)	0.498
rm15—My appetite is not very good because of my back.	0.460 *** (0.072)		0.212	0.254 ** **(0.102)**	0.241 * (0.094)	0.189
**rm18—I sleep less well because of my back.**	0.777 *** (0.043)		0.603	0.567 *** **(0.068)**	0.258 ** (0.083)	0.547
**rm20—I sit down for most of the day because of my back**.	0.513 *** (0.066)		0.263	0.318 *** **(0.078)**	0.227 ** (0.079)	0.231
rm22—Because of back pain, I am more irritable and bad tempered with people than usual.	0.754 *** (0.053)		0.569	0.608 *** **(0.068)**	0.189 * (0.083)	0.531
n1-I’m tired all the time.	0.618 *** (0.073)		0.382	0.455 *** **(0.088)**	0.192 (0.101)	0.340
n2-I have pain at night.	0.675 *** (0.052)		0.456	0.590 *** **(0.074)**	0.122 (0.087)	0.442
n3—Things are getting me down.	0.732 *** (0.044)		0.535	0.829 *** **(0.055)**	−0.081 (0.078)	0.620
**n4—I have unbearable pain.**	0.663 *** (0.061)		0.440	0.437 *** **(0.078)**	0.270 *** (0.078)	0.393
n5—I take pills to help me sleep.	0.519 *** (0.064)		0.269	0.323 *** **(0.092)**	0.230 ** (0.086)	0.239
**n6—I’ve forgotten what it’s like to enjoy myself.**	0.760 *** (0.045)		0.578	0.509 *** **(0.069)**	0.299 *** (0.066)	0.516
n7-I’m feeling on edge.	0.774 *** (0.042)		0.600	0.849 *** **(0.045)**	−0.056 (0.066)	0.671
n9—I feel lonely.	0.850 *** (0.032)		0.723	0.954 *** **(0.033)**	−0.117 (0.056)	0.802
n13-I’m waking up in the early hours of the morning.	0.519 *** (0.069)		0.269	0.419 *** **(0.078)**	0.128 (0.086)	0.250
n15-I’m finding it hard to make contact with people.	0.848 *** (0.041)		0.719	0.869 *** **(0.046)**	0.002 (0.077)	0.757
n16—The days seem to drag.	0.841*** (0.034)		0.707	0.864 *** **(0.043)**	−0.004 (0.062)	0.743
n20-I lose my temper easily these days.	0.684 *** (0.05)		0.467	0.858 *** **(0.048)**	−0.173 * (0.073)	0.603
n21-I feel there is nobody that I am close to.	0.827 *** (0.037)		0.683	0.962 *** **(0.036)**	−0.161 * (0.067)	0.783
n22-I lie awake for most of the night.	0.844 *** (0.032)		0.712	0.776*** **(0.053)**	0.107 (0.072)	0.705
n23-I feel as if I’m losing control.	0.728 *** (0.049)		0.530	0.618 *** **(0.06)**	0.149 * (0.073)	0.505
**n28—I’m in constant pain**	0.819 *** (0.045)		0.671	0.468 *** **(0.071)**	0.393 *** (0.076)	0.574
n29—It takes me a long time to get to sleep.	0.686 *** (0.05)		0.470	0.659 *** **(0.061)**	0.058 (0.079)	0.479
n30—I feel I am a burden to people.	0.740 *** (0.047)		0.548	0.661 *** **(0.059)**	0.119 (0.068)	0.538
n31—Worry is keeping me awake at night.	0.735 *** (0.043)		0.541	0.820*** **(0.05)**	−0.071 (0.069)	0.613
n32—I feel that life is not worth living.	0.711*** (0.052)		0.506	0.885 *** **(0.047)**	−0.181 * (0.075)	0.641
n33—I sleep badly at night.	0.870 *** (0.028)		0.757	0.895 *** **(0.042)**	0.005 (0.062)	0.807
n34-I’m finding it hard to get along with people.	0.736 *** (0.06)		0.541	0.832 *** **(0.051)**	−0.081 (0.078)	0.626
n37—I wake up feeling depressed.	0.718 *** (0.046)		0.515	0.808 *** **(0.051)**	−0.072 (0.069)	0.594
wd2.1—standing for long periods such as 30 min?		0.601 *** (0.046)	0.361	−0.022 (0.059)	0.627 *** **(0.048)**	0.378
wd2.2—standing up from sitting down?		0.703 *** (0.034)	0.495	0.045 (0.066)	0.678 *** **(0.045)**	0.495
wd2.3—moving around inside your home?		0.625 *** (0.042)	0.391	−0.058 (0.058)	0.680 *** **(0.046)**	0.422
wd2.4—getting out of your home?		0.750 *** (0.03)	0.563	−0.031 (0.055)	0.780 *** **(0.039)**	0.583
wd2.5—walking a long distance such as a kilometer (or equivalent)?		0.670 *** (0.035)	0.449	−0.08 (0.061)	0.738 *** **(0.047)**	0.486
wd3.1—washing your whole body?		0.669 *** (0.042)	0.448	0.079 (0.063)	0.616 *** **(0.052)**	0.439
wd3.2—getting dressed?		0.660 *** (0.039)	0.436	−0.054 (0.062)	0.707 *** **(0.044)**	0.462
wd3.3—eating?		0.562 *** (0.052)	0.316	0.143 (0.086)	0.457 *** **(0.071)**	0.300
wd3.4—staying by yourself for a few days?		0.502 *** (0.053)	0.252	0.088 (0.071)	0.440 *** **(0.06)**	0.244
**wd5.2—taking care of your household responsibilities?**		0.806 *** (0.024)	0.649	−0.273*** (0.066)	0.969 *** **(0.037)**	0.724
**wd5.3—doing most important household tasks well?**		0.816 *** (0.024)	0.665	−0.301*** (0.063)	0.992 *** **(0.032)**	0.748
**wd5.4—getting all the household work done that you needed to do?**		0.900 *** (0.015)	0.811	−0.378*** (0.064)	1.088 *** **(0.032)**	0.876
**wd5.5—getting your household work done as quickly as needed?**		0.882 *** (0.017)	0.778	−0.371*** (0.065)	1.067 *** **(0.03)**	0.843
wd6.1—In the last 30 days, how much of a problem did you have in joining in community activities (for example, festivities, religious or other activities) in the same way as anyone else can		0.624 *** (0.042)	0.390	0.051 (0.074)	0.595 *** **(0.06)**	0.389
wd6.2—In the last 30 days, how much of a problem did you have because of barriers or hindrances in the world around you?		0.516 *** (0.049)	0.266	0.128 (0.076)	0.419 *** **(0.066)**	0.251
wd6.4—In the last 30 days, how much time did you spend on your health condition, or its consequences?		0.214 ** (0.065)	0.046	0.049 (0.071)	0.180 ** **(0.066)**	0.044
**wd6.5—In the last 30 days, how much have you been emotionally affected by your health condition?**		0.659 *** (0.042)	0.434	0.274 *** (0.057)	0.460 *** **(0.055)**	0.425
wd6.6—In the last 30 days, how much has your health been a drain on the financial resources of you or your family?		0.226 ** (0.068)	0.051	0.096 (0.096)	**0.149** **(0.093)**	0.047
wd6.7—In the last 30 days, how much of a problem did your family have because of your health problems?		0.443 *** (0.053)	0.196	0.177 * (0.076)	0.308 *** **(0.069)**	0.186
wd6.8—In the last 30 days, how much of a problem did you have in doing things by yourself for relaxation or pleasure?		0.680 *** (0.037)	0.462	−0.031 (0.055)	0.711 *** **(0.041)**	0.482
**o1-Pain Intensity**		0.506 *** (0.042)	0.256	0.177 ** (0.064)	0.381 *** **(0.055)**	0.251
o2—Personal Care (Washing, Dressing)		0.525 *** (0.043)	0.275	0.058 (0.067)	0.486 *** **(0.06)**	0.271
o3-Lifting		0.369 *** (0.057)	0.136	−0.122 (0.073)	0.468 *** **(0.058)**	0.171
o4-Walking		0.628 *** (0.04)	0.394	−0.049 (0.064)	0.672 *** **(0.049)**	0.419
o5-Sitting		0.513 *** (0.047)	0.263	0.160 * (0.064)	0.394 *** **(0.063)**	0.250
o6-Standing		0.630 *** (0.038)	0.397	−0.055 (0.059)	0.678 *** **(0.047)**	0.422
o9-Social Life		0.540 *** (0.044)	0.292	0.070 (0.068)	0.495 *** **(0.059)**	0.288
o10-Travelling		0.612 *** (0.045)	0.375	0.060 (0.068)	0.575 *** **(0.059)**	0.372
rm1—I stay at home most of the time because of my back.		0.714 *** (0.05)	0.510	0.131 (0.082)	0.624 *** **(0.066)**	0.496
**rm2—I change position frequently to try to get my back comfortable.**		0.746 *** (0.083)	0.556	0.292 ** (0.099)	0.537 *** **(0.115)**	0.545
rm3—I walk more slowly than usual because of my back.		0.833 *** (0.043)	0.693	0.054 (0.074)	0.804 *** **(0.066)**	0.697
**rm4—Because of my back, I am not doing any jobs that I usually do around the house.**		0.694 *** (0.057)	0.482	0.281 ** (0.085)	0.477 *** **(0.08)**	0.454
rm5—Because of my back, I use a handrail to get upstairs.		0.834 *** (0.046)	0.696	0.077 (0.09)	0.786 *** **(0.067)**	0.691
rm6—Because of my back, I lie down to rest more often.		0.576 *** (0.076)	0.332	0.071 (0.096)	0.530 *** **(0.093)**	0.327
**rm7—Because of my back, I have to hold on to something to get out of an easy chair.**		0.661 *** (0.053)	0.437	0.227 ** (0.087)	0.490 *** **(0.083)**	0.413
rm8—Because of my back, I try to get other people to do things for me.		0.500 *** (0.067)	0.250	0.145 (0.088)	0.388 *** **(0.082)**	0.233
**rm9—I get dressed more slowly than usual because of my back.**		0.691 *** (0.052)	0.477	0.214 ** (0.079)	0.532 *** **(0.074)**	0.454
rm10—I only stand up for short periods of time because of my back.		0.815 *** (0.048)	0.664	−0.128 (0.09)	0.912 *** **(0.067)**	0.721
**rm11—Because of my back, I try not to bend or kneel down.**		0.835 *** (0.063)	0.697	0.345 *** (0.083)	0.589 *** **(0.089)**	0.689
**rm12—I find it difficult to get out of a chair because of my back.**		0.772 *** (0.041)	0.596	0.239 ** (0.079)	0.597 *** **(0.074)**	0.570
**rm14—I find it difficult to turn over in bed because of my back.**		0.777 *** (0.047)	0.604	0.315 *** (0.08)	0.543 *** **(0.077)**	0.582
rm16—I have trouble putting on my sock (or stockings) because of the pain in my back.		0.660 *** (0.055)	0.435	0.004 (0.082)	0.663 *** **(0.074)**	0.443
rm17—I can only walk short distances because of my back pain.		0.779 *** (0.044)	0.607	−0.015 (0.068)	0.799 *** **(0.049)**	0.627
rm19—Because of my back pain, I get dressed with the help of someone else.		0.574 *** (0.082)	0.330	−0.098 (0.137)	0.664 *** **(0.115)**	0.380
**rm21—I avoid heavy jobs around the house because of my back.**		0.630 *** (0.069)	0.397	0.229 * (0.101)	0.457 *** **(0.091)**	0.376
rm23—Because of my back, I go upstairs more slowly than usual.		0.826 *** (0.047)	0.683	0.078 (0.104)	0.777 *** **(0.087)**	0.677
rm24—I stay in bed most of the time because of my back.		0.501 *** (0.057)	0.251	0.120 (0.099)	0.412 *** **(0.086)**	0.238
**n8-I find it painful to change position.**		0.786 *** (0.047)	0.617	0.227 ** (0.078)	0.627 *** **(0.078)**	0.600
n11—I find it hard to bend.		0.770 *** (0.054)	0.593	0.100 (0.094)	0.706*** **(0.075)**	0.585
**n12-Everything is an effort.**		0.761 *** (0.044)	0.580	0.220 ** (0.068)	0.605 *** **(0.068)**	0.561
n17—I have trouble getting up and down stairs and steps.		0.886 *** (0.037)	0.785	−0.062 (0.093)	0.938 *** **(0.057)**	0.820
n18—I find it hard to reach for things.		0.852 *** (0.043)	0.725	0.144 (0.083)	0.756 *** **(0.070)**	0.712
n19—I’m in pain when I walk.		0.757 *** (0.055)	0.573	0.129 (0.094)	0.670 *** **(0.075)**	0.560
n24—I’m in pain when I’m standing.		0.724 *** (0.065)	0.524	−0.017 (0.138)	0.742 *** **(0.116)**	0.536
n25—I find it hard to get dressed by myself.		0.589 *** (0.062)	0.347	0.050 (0.079)	0.559 *** **(0.065)**	0.345
**n26—I soon run out of energy.**		0.684 *** (0.068)	0.467	0.316 ** (0.103)	0.454 *** **(0.111)**	0.462
n27—I find it hard to stand for long (e.g., at the kitchen sink, waiting in a line).		0.774 *** (0.066)	0.599	−0.119 (0.157)	0.861 *** **(0.114)**	0.643
n36—I’m in pain when going up or down stairs.		0.829 *** (0.047)	0.688	−0.056 (0.102)	0.880 *** **(0.066)**	0.723
n38—I’m in pain when I’m sitting.		0.583 *** (0.069)	0.339	−0.028 (0.081)	0.614 *** **(0.078)**	0.360

Abbreviations: wd: WHODAS-II, o: Oswestry, rm: Roland Morris, n: NHP. “In the last 30 days, how much difficulty…” statement is valid for all items coded as wd. The loadings for the target factors of items were given in bold. Items loaded on both factors statistically and clinically are written in bold. *** *p* < 0.001; ** *p* < 0.01; * *p* < 0.05.

## Data Availability

The datasets generated and/or analyzed during the current study are available from the corresponding author upon reasonable request.

## Supplementary Materials

The following supporting information can be downloaded at: https://www.mdpi.com/article/10.3390/jcm14238551/s1, File S1: Mplus syntax used for the ESEM analyses.

## References

[1] Vlaeyen J.W.S., Maher C.G., Wiech K., Van Zundert J., Meloto C.B., Diatchenko L., Battié M.C., Goossens M., Koes B., Linton S.J. (2018). Low back pain. Nat. Rev. Dis. Primers.

[2] Chiarotto A., Koes B.W. (2022). Nonspecific low back pain. N. Engl. J. Med..

[3] GBD 2021 Low Back Pain Collaborators (2023). Global, regional, and national burden of low back pain, 1990–2020, its attributable risk factors, and projections to 2050: A systematic analysis of the Global Burden of Disease Study 2021. Lancet Rheumatol..

[4] Garg A., Pathak H., Churyukanov M.V., Uppin R.B., Slobodin T.M. (2020). Low back pain: Critical assessment of various scales. Eur. Spine J..

[5] Elhan A.H., Öztuna D., Kutlay Ş., Küçükdeveci A.A., Tennant A. (2008). An initial application of computerized adaptive testing (CAT) for measuring disability in patients with low back pain. BMC Musculoskelet. Disord..

[6] Wong J.J., Côté P., Tricco A.C., Rosella L.C. (2019). Examining the effects of low back pain and mental health symptoms on healthcare utilisation and costs: A protocol for a population-based cohort study. BMJ Open.

[7] Froud R., Patterson S., Eldridge S., Seale C., Pincus T., Rajendran D., Fossum C., Underwood M. (2014). A systematic review and meta-synthesis of the impact of low back pain on people’s lives. BMC Musculoskelet. Disord..

[8] Chiarotto A., Ostelo R.W., Boers M., Terwee C.B. (2018). A systematic review highlights the need to investigate the content validity of patient-reported outcome measures for physical functioning in patients with low back pain. J. Clin. Epidemiol..

[9] Kamper S.J., Logan G., Copsey B., Thompson J., Machado G.C., Abdel-Shaheed C., Williams C.M., Maher C.G., Hall A.M. (2020). What is usual care for low back pain? A systematic review of health care provided to patients with low back pain in family practice and emergency departments. Pain.

[10] Reise S.P., Morizot J., Hays R.D. (2007). The role of the bifactor model in resolving dimensionality issues in health outcomes measures. Qual. Life Res..

[11] Morin A.J., Arens A.K., Marsh H.W. (2016). A bifactor exploratory structural equation modeling framework for the identification of distinct sources of construct-relevant psychometric multidimensionality. Struct. Equ. Model..

[12] Asparouhov T., Muthén B. (2009). Exploratory structural equation modeling. Struct. Equ. Model..

[13] Marsh H.W., Morin A.J., Parker P.D., Kaur G. (2014). Exploratory structural equation modeling: An integration of the best features of exploratory and confirmatory factor analysis. Annu. Rev. Clin. Psychol..

[14] Melton B.L., Moqbel M., Kanaan S., Sharma N.K. (2016). Structural equation model of disability in low back pain. Spine.

[15] Öztuna D. (2008). An Application of Computerized Adaptive Testing in the Evaluation of Disability in Musculoskeletal Disorders. Ph.D. Thesis.

[16] Hayme S. (2021). Evaluation of Measurement Tools’ Dimensionality Structure with the Methods Based on Structural Equation Modeling. Ph.D. Thesis.

[17] World Health Organization (2001). ICF: International Classification of Functioning, Disability and Health.

[18] Cieza A., Stucki G., Weigl M., Disler P., Jackel W., van der Linden S., Kostanjsek N., de Bie R., Stucki G., Jäckel W. (2004). ICF Core Sets for low back pain. J. Rehabil. Med..

[19] Mousavi S.J., Parnianpour M., Mehdian H., Montazeri A., Mobini B. (2006). The Oswestry disability index, the Roland-Morris disability questionnaire, and the Quebec back pain disability scale: Translation and validation studies of the Iranian versions. Spine.

[20] Fairbank J.C., Pynsent P.B. (2000). The Oswestry disability index. Spine.

[21] Roland M., Fairbank J. (2000). The Roland–Morris disability questionnaire and the Oswestry disability questionnaire. Spine.

[22] Morin A.J., Myers N.D., Lee S. (2020). Modern factor analytic techniques: Bifactor models, exploratory structural equation modeling (ESEM), and bifactor-ESEM. Handbook of Sport Psychology.

[23] Hayme S. (2015). The Evaluation of Measurement Equivalence via Multiple Group Analysis and Rasch Analysis. Master’s Thesis.

[24] Brown T.A. (2015). Confirmatory Factor Analysis for Applied Research.

[25] Rodriguez A., Reise S.P., Haviland M.G. (2016). Applying bifactor statistical indices in the evaluation of psychological measures. J. Pers. Assess..

[26] Jenks A., Hoekstra T., van Tulder M., Ostelo R.W., Rubinstein S.M., Chiarotto A. (2022). Roland-Morris Disability Questionnaire, Oswestry Disability Index, and Quebec Back Pain Disability Scale: Which has superior measurement properties in older adults with low back pain?. J. Orthop. Sports Phys. Ther..

[27] Wong A.Y., Forss K.S., Jakobsson J., Schoeb V., Kumlien C., Borglin G. (2018). Older adult’s experience of chronic low back pain and its implications on their daily life: Study protocol of a systematic review of qualitative research. Syst. Rev..

[28] Fresno A., Arias V., Núñez D., Spencer R., Ramos N., Espinoza C., Bravo P., Arriagada J., Brunet A. (2020). Using exploratory structural equation modeling (ESEM) to examine the internal structure of posttraumatic stress disorder symptoms. Span. J. Psychol..

[29] Van Der Roer N., Ostelo R.W., Bekkering G.E., Van Tulder M.W., De Vet H.C. (2006). Minimal clinically important change for pain intensity, functional status, and general health status in patients with nonspecific low back pain. Spine.

[30] Terwee C.B., Prinsen C.A.C., Chiarotto A., Westerman M.J., Patrick D.L., Alonso J., Bouter L.M., de Vet H.C.W., Mokkink L.B. (2018). COSMIN methodology for evaluating the content validity of patient-reported outcome measures: A Delphi study. Qual. Life Res..

[31] Morin A.J., Arens A.K., Tran A., Caci H. (2016). Exploring sources of construct-relevant multidimensionality in psychiatric measurement: A tutorial and illustration using the Composite Scale of Morningness. Int. J. Methods Psychiatr. Res..

[32] Mescouto K., Olson R.E., Hodges P.W., Setchell J. (2022). A critical review of the biopsychosocial model of low back pain care: Time for a new approach?. Disabil. Rehabil..

