# Supplementary material for: Re-Examining the Dimensionality of a Disability Assessment Tool Using Exploratory Structural Equation Modeling (ESEM): A Preliminary Study in Low Back Pain
Source: J Clin Med. 2025 Dec 2;14(23):8551. doi: 10.3390/jcm14238551 (PMC12693373; doi:10.3390/jcm14238551)
Supplement: Supplementary file 1 [file jcm-14-08551-s001.zip › jcm-3975134-supplementary.pdf]

```
TITLE: ESEM

DATA:
  FILE IS lbp.dat;
VARIABLE:
  NAMES ARE u1-u102;
  USEVARIABLE ARE U1-U73 U75-U77 u79-u98 u100-u102;
  CATEGORICAL ARE U1-U73 U75-U77 u79-u98 u100-u102;
  MISSING ARE all (9);
ANALYSIS:
  ESTIMATOR = WLSMV;
  ROTATION = TARGET(OBLIQUE);

MODEL:
  F1 BY U1-U6 U7-U15~0 U16-U19 U20-U25~0 U26 U27-u30~0 U31-U37~0 U38
     U39-U52~0 U53 U54~0 U55 U56-U57~0 U58 U59~0 U60 U61~0 U62
     U63-U64~0 U65-U71 U72~0 U73 U75-U76~0 U77 u79-U80 U81-U83~0
     U84-U87 U88-U91~0 U92-U98 U100~0 U101 U102~0 (*1);
  F2 BY U1-U6~0 U7-U15 U16-U19~0 U20-U25 U26~0 U27-u30 U31-U37 U38~0
     U39-U52 U53~0 U54 U55~0 U56-U57 U58~0 U59 U60~0 U61 U62~0
     U63-U64 U65-U71~0 U72 U73~0 U75-U76 U77~0 u79-U80~0 U81-U83
     U84-U87~0 U88-U91 U92-U98~0 U100 U101~0 U102 (*1);

OUTPUT:
  SAMPSTAT STANDARDIZED RESIDUAL TECH4;

SAVEDATA:
FILE IS fsscores.dat;
SAVE = FSCORES;
```
